# MicroRNAs networks in thyroid cancers: focus on miRNAs related to the fascin

**DOI:** 10.1186/2251-6581-12-31

**Published:** 2013-07-01

**Authors:** Hilda Samimi, Majid Zaki dizaji, Mohsen Ghadami, Abolhasan Shahzadeh fazeli, Patricia Khashayar, Masoud Soleimani, Bagher Larijani, Vahid Haghpanah

**Affiliations:** 1 Science and Culture University, Tehran, Iran; 2 Endocrinology and Metabolism Research Center, Endocrinology and Metabolism Research Institute, Tehran University of Medical Sciences, 5th floor, Dr. Shariati Hospital, North Kargar Ave, Tehran, Iran; 3 Department of Medical Genetic, Tehran University of Medical Sciences, Tehran, Iran; 4 Department of Hematology, Faculty of Medical Science, Tarbiat Modares University, Tehran, Iran

**Keywords:** Thyroid Cancer, microRNA, Oncomir, Fascin

## Abstract

miRNAs are non coding ribonucleic acids which are protected with respect to evolution, and have a length of 18–25 nucleotides. microRNAs control the gene expression after transcription, through mRNA destruction or translation processing, and therefore participate in arrangement of the physiologic and pathologic cellular processes; They also may act as oncogene or tumor suppressors. Altered expression of a number of microRNAs is reported in process of progression and metastasis of thyroid cancers. Therefore, identification of these microRNAs may shed a light to oncogenesis pathway of thyroid cancers and their metastasis. In addition, microRNAs might apply as potential biological markers in diagnosis and treatment of thyroid cancers. The changes made in miRNAs profile of thyroid cancers are reviewed in this paper.

## Introduction

Despite the fact that thyroid malignances are ranked 14th among all kinds of cancers, and affect only 1- 2% of all cancer sufferers, but they are considered as the most prevalent cancer of the endocrinology system. The annual occurrence of such cancer ranges from 0.5 to 10% among 100000 persons in different parts of the world [1–3]; women are believed to be 2–4 times more likely to develop the condition [4]. Various studies show that about 3-5% of the affected subjects have positive family histories of the cancer [5, 6]. In addition to the effect of sex and genetic factors, the size of the body [7, 8], race [9], geographical distribution [10] and the amount of iodine intake [11] affect the rate of developing thyroid cancers. Thyroid malignances may break out at any age; nevertheless, the majority of the cases are aged over 30. It is specified that with aging, the invasion rate of such cancers increase [2, 12].

Pathological analysis of thyroid cancer demonstrates that four types of the condition are more prevalent: papillary, follicular, anaplastic and medullary thyroid carcinoma. The first three categories origin from follicular cells, whereas medullary carcinoma derives from para-follicular cells (C-cells) [3, 13]. Recent studies conducted in this field revealed that the gradual evolution of natural cells to neoplastic ones in the process of tumor development is the result of sequential genetic events, from among which changes in miRNAs profile is the main focus of the present study.

## miRNAs

In 1993, the first miRNA, also known as Lin-4 (Lineage-4), was discovered in C. Elegans; it was, however, not earlier than 2001 that Let-7 was discovered. At that time, the importance of such molecules as a biological regulator had not been determined [14].

Micro-RNAs (miRNAs) are single strand 18-25-nucleotide RNAs, which are transcribed by RNA Polymerase II from miRNA genes. They are however not translated into proteins. As the negative regulators of gene expression, they bind to 3'UTR of the Target mRNA, and prevent the translation process through destructing or blocking the mRNA. miRNAs are non-encoder endogenous RNAs, that apart from negative regulation of encoder protein genes, regulate various cellular processes such as reproduction, proliferation, difference, cell survival and carcinogenesis mechanisms [15]. Mechanism of miRNAs in the regulation of gene expression is the same as that of siRNAs, although, they are completely different. It is estimated that miRNA genes account for 2-5% of the human genome [16]. Up to now, more than 1800 types of miRNAs are recognized in plants, animals and even in viruses. It is estimated that human’s genome includes 800 to 1000 miRNAs, most of which are only found in human beings [17]. Most of the miRNAgenes, located within the intron and exon of the encoding genes of various proteins and may be transcribed the polycistron. For instance, miR-221 and miR-222 are located on chromosome X and their deregulated expression is shown in some carcinomas such as thyroid cancer [18].

Approximately, each miRNA controls the expression of more than 200 Genes. Many miRNAs have special tissue expression, which may be higher or lower than that of normal tissues [19]. In other words, altered expression of certain miRNAs is linked with some cancers, including various kinds of thyroid cancer [18]. Although, the exact function of many miRNAs is not well distinguished, many believe determination of important miRNAs in cancerous tissues may help improve the understanding of the regulation of gene expression in various types of cancer. Cancer occurs when the regulation, proliferation and differentiation process is altered. The cancers that are insensitive to growth stimulating and suppressing signals, have unlimited proliferation and angiogenesis potential, are preserved from apoptosis, escape the immune system, have genome instability and metastasis ability may become malignant [20–23] (Table 1).

**Table 1:** **Role of miRNAs in the development of certain types of cancer**[24]

Main characteristics of Cancer	Function	miRNAs
Insensitivity to stimulating and suppressing signals of growth	• Growth stimulation	• miR-21,-17 Cluster,-221,-222
	• Growth suppression	• miR-519,-146a,Let-7
Escape from programmed cell death (Apoptosis)	• Apoptosis stimulation	• miR-34Cluster,-29,-15,-10
	• Apoptosis suppression	• miR-290,-24,-34a
Unlimited proliferation potential	• Immortality and aging control	• miR-290,-24,-34a
Angiogenesis induction	• Angiogenesis stimulation	• MiR-17-92Cluster,-378,-996,-27b,-130,-126,Let-7f
	• Angiogenesis suppression	
		• miR-15,-16,-20a,-20b
Escape from immune system	• Escape from immune response	• miR-17-92Cluster,-155,-20a,-93,-106b,-372,-373,-520c
Metastasis	• Metastasis stimulation	• miR-10b,-21,-373,-520c,-155,--335,-206,-126,Let-7
	• Metastasis suppression	
		• miR-146a,-101,-200
Genome instability	• Genome instability induction	• miR-16-1,-17,-20A,-15

Altered expression of miRNAs through decreased expression of certain genes involved in cell proliferation and survival may result in cancer. This however does not suggest that miRNAs are directly involved in tumorigenesis and the development of cancer as the available studies have failed to show whether altered expression of mRNAs is the consequence of cancer or malignancies are secondary to altered expression. Nonetheless, certain changes that occur in cancerous cells can affect miRNAs expression directly or indirectly [25].

In some types of cancers, such as thyroid neoplasia, miRNAs are deregulated. Altered expression of miRNAs plays an important role in tumorigenesis. Special subsets of miRNAs in certain tumors have increased or reduced expression. Increased expression of miRNAs may result in reduced expression of tumor suppressor genes, and their decreased expression may activate proto-oncogenes. The number of genes, considered as the target of miRNAs and involved in cancer, is increasing very fast [19]. For instance, let-7 is a negative regulator of RAS [25], whereas miR-221 and miR-222 are the negative regulators of KIT Receptor [26]. CDKN1B, an important control factor in cell cycle, is linked with cell development in S Phase of the cycle) [27]. miR-16-1 and miR-15a may cause reduced expression of BCL2, which is involved in apoptosis [28]. Such miRNAs, also known as oncomirs, are present in various types of cancer. Their genes are located on the loci on which deletion, duplication and point mutation has occurred [29, 30]. Some of microRNAs act through reducing the expression of tumor suppressing genes, cell differentiation regulatory genes and apoptosis. Others, on the other hand, act through targeting proto-oncogenic mRNAs and silencing such mRNAs, thus, lower the risk of these cells becoming cancerous [31]. Altered expression of some miRNAs may cause cells to become cancerous, affect cell growth through interfering with the regulation of cell cycle. miRNAs are the most important factors in the regulation of programmed cell death during tumorigenesis; the survival of cancerous cells, on the other hand, are influenced by changing the expression of such micro-RNAs.

Cancerous cells become immortal by protecting their telomeres through positive regulation of telomerase and telomere preservation. Any change in the expression of miRNAs interferes with high-level telomerase activity in tumors [32]. Deregulatory mechanisms of miRNAs in tumor tissues are not completely determined [33, 34].

Recently, adding other molecular markers such as miRNAs to the mutant panels has improved the sensitivity of cancer diagnosis. miRNAs are involved in the pathogenesis of certain cancers, such as thyroid neoplasia. Not much is known about the clinical and pathological properties of the disease and expression of special miRNAs; however altered miRNA expression is reported in cancerous thyroid tissues compared with healthy cells. In other words, increased miRNA expression is reported in 32% of thyroid malignances, whereas 38% of them present reduced expression [35] (Table 2).

**Table 2:** **Some of the miRNAs involved in various kinds of thyroid cancers**

Thyroid tumor	Down-regulation	Up-regulation	References
PapillaryCarcinoma	miR-146,-221,-222,-21,-181a, -155,-213,-181b,-31,-172,-34a,-223,-224,-187,-146b,-220	miR-26a-1,-345,-138,-319,-218,-300,-292,-30c	[35–38]
Follicular Carcinoma	miR-197,-346,-187,-221,-222,-224,-203,-183,-339,-31		[35]
Anaplastic Carcinoma	miR-302c,-205,-137,-187,-214,-155,-224,-222,-221	miR-30d,-125b,-26a,-30a,-5p	[35, 36]
Medullary Carcinoma	miR-323,-370,-129,-137,-10a,-124a,-224,-127,-9,-154		[35]

Such information suggests the role of deregulated expression of miRNAs in the transformation of thyroid cells. For instance, increased miR-187 expression is reported in tumors with RET/PTC gene rearrangement, whereas that of miR-221 and miR-22 is observed in tumors positive for BRAF and RAS mutations as well as papillary carcinomas with unclear mutation. Increased miR-146b expression is seen in tumors with RAS mutation. Such information can help facilitate detecting malignant tumors in FNA (Fine Needle Aspiration) samples with high sensitivity even when molecular tests have failed to report any mutations [35–40].

## microRNAs in Epithelial Mesenchymal Transition (EMT)

Metastasis is a complicated dynamic biological event, subject to the separation of cancerous cells from adjacent tissue. Indeed, metastasis increases the risk of death secondary to cancer. There are different special mechanisms for the metastatic processin various cancerous cells. Shifting the focus to the studies aiming to recognize the reason behind the changes noted in the expression of suppressor genes or metastasis activators can pave the way to better understand different aspects of such phenomenon, and thus develop effective ways to prevent cancer. EMT is one of the most important way of the metastatic process [41]. The miRNAs-200 family islately considered as strong regulators of EMT, as they affect the balance between EMT and MET its reversed process [42]. This family consists of five members divided into two categories of 200a/200b/429 and 200c/141 [43]. miRNA-200c regulates EMT through suppressing ZEB1/2 (transcription suppressors of E-cadherin). This miRNAcan suppress Bmi-1, which is a polycomb protein responsible for the preservation of its essential characteristics, in healthy and cancerous stem cells. Recent studies confirm the role of miRNA-200c in the regulation of EMT [44].

The invasion and metastasis of tumor cells is a major cause of mortality in cancer patients. Fascin is another protein involved in EMT process. Fascins are globular proteins of approximately 55 kDa composed of four tandem fascin domains, each of which corresponds structurally to a β-trefoil fold. Fascin1, an actin-bundling protein, has been demonstrated to be critical for filopodia formation and thus is believed to be vital in movement including and cell migration. The formation of such structures is associated with increased risk of invasion and metastasis [45]. Immunohistochemistry (IHC), tissue microarray (TMA) study and qRT- PCR of various cancers confirms increased expression of Fascin-1,especially in metastatic lung and breast neoplasia [46]. miRNAs are important factors involved in the regulation of fascin expression rate. Different studies have revealed the role of 145, 133a, 133b and miRNA-146a in increased expression of fascin [47].

## Conclusion

More research is needed to clarify the regulatory mechanisms of microRNAs and their role in the development of thyroid cancers. The study of target microRNA molecules along with their effects on signaling pathways and metastasis may cause better understanding of such mechanisms. Furthermore, microRNAs can pave the way for the development of novel treatment modalities for thyroid cancers; however, these options are associated with certain impediments and problems. For instance, microRNAs control the expression of certain target genes; therefore, change in any of such genes may target other genes rather than the main ones. On the other hand, several microRNAs may control a single gene by and thus, any change in the expression of even one of this microRNA may not be an effective treatment in this regard. Also, transfer of antisense oligonucleotides for decreased expression of microRNAs, which are involved in cancer, may affect non-targeted microRNAs, and therefore result in undesirable effects in the process of the disease. Considering the above mentioned facts, in order to benefit from microRNAs, increase their usefulness, and minimize the undesirable effects arising from interconnection of antisense oligonucleotides to the non-target microRNAs, further studies are needed.

## Abbreviations

miR: microRNA
FNA: Fine Needle Aspiration
EMT: Epithelial Mesenchymal Transition
IHC: Immunohistochemistry
TMA: Tissue microarray
qRT-PCR: Quantitative Real Time – Polymerase Chain Reaction.
